# The Synergistic Armory: A Global Genome-Wide Association Study Reveals the Integrated Mechanisms of Azithromycin Resistance in *Neisseria gonorrhoeae*

**DOI:** 10.3390/ijms27052258

**Published:** 2026-02-27

**Authors:** Boris Shaskolskiy, Konstantin Tutaev, Dmitry Kravtsov, Ilya Kandinov, Dmitry Gryadunov

**Affiliations:** Engelhardt Institute of Molecular Biology, Russian Academy of Sciences, 119991 Moscow, Russia; konstantintutaev@yandex.ru (K.T.); solo13.37@yandex.ru (D.K.); kandinov@biochip.ru (I.K.); grad@biochip.ru (D.G.)

**Keywords:** *Neisseria gonorrhoeae*, azithromycin resistance, genome-wide association study, phylogenetic analysis

## Abstract

Azithromycin remains an important agent in gonorrhea treatment, yet resistance is a growing global threat. To comprehensively define its genetic basis, we performed a large-scale genome-wide association study of 14,727 *Neisseria gonorrhoeae* genomes with linked azithromycin MICs from 66 countries. We identified 113 genetic variants significantly associated with elevated MICs. Beyond well-known mutations in 23S rRNA (A2059G, C2611T) and *mtrCDE* operon, we uncovered a broad repertoire of potential resistance determinants, including multiple amino acid substitutions in 16 ribosomal proteins (e.g., L2, L4, L13, L23) forming the nascent peptide exit tunnel (NPET), and porin PorB alterations (G120K, A121D/N). Systematic pairwise analysis revealed extensive synergistic interactions, particularly between variants affecting drug influx/efflux (PorB, MtrCDE) and ribosomal target affinity. Phylogenetic analysis identified successful, globally circulating lineages employing distinct resistance strategies: NPET-dominated, 23S rRNA-associated, and porin/efflux-mediated. Our findings demonstrate that azithromycin resistance is a polygenic trait shaped by functional complementarity and epistasis between target modification, membrane permeability, and efflux. This integrated model is essential for accurate resistance prediction from genomic data and highlights key lineages for focused surveillance.

## 1. Introduction

*Neisseria gonorrhoeae* is an obligate human pathogen that primarily colonizes the mucosal epithelium of the urogenital tract but can also infect the rectum, pharynx, and conjunctiva [1]. Up to half of all infections remain asymptomatic, contributing to delayed diagnosis and increasing the risk of complications such as pelvic inflammatory disease, infertility, epididymitis, and disseminated gonococcal infection affecting the joints, heart, and other organs [1]. According to WHO estimates, 82 million new gonorrhea cases occurred globally in 2020 among individuals aged 15–49 years [2].

The updated 2023 WHO guidelines for empirical gonorrhea treatment recommend ceftriaxone, cefixime, spectinomycin, gentamicin, and azithromycin [3]. Azithromycin is included in combination regimens when local susceptibility patterns support its use and is considered an alternative option when β-lactams are contraindicated [3].

Azithromycin is highly lipophilic and demonstrates an oral bioavailability of approximately 37% due to extensive first-pass hepatic metabolism [4]. Its long terminal half-life (68–96 h) enables broad tissue penetration and pronounced intracellular accumulation, particularly within phagocytic cells such as neutrophils and macrophages, where concentrations reach 50–100 times those in plasma [5].

Macrolides bind within the nascent peptide exit tunnel (NPET) of the 50S ribosomal subunit, positioned approximately 10–15 Å from the peptidyl transferase center (PTC). Their primary interactions involve the macrolactone ring and the desosamine moiety, which contact nucleotides A2058, A2059, and C2611 in domain V of the 23S rRNA that line the tunnel wall [6,7]. A key feature of macrolide action is that these antibiotics do not fully occlude the NPET; instead, they narrow its lumen, leaving an aperture of ~5–6 Å that still permits the passage of certain elongating peptides. This structural arrangement explains why macrolides do not cause complete translational arrest but rather act in a context-dependent manner, stalling ribosomes only during the synthesis of peptides containing sequence-specific macrolide-arrest motifs (MAMs), such as +X+ (Arg/Lys-X-Arg/Lys) or X-Asp-Lys [8,9,10,11]. When such motifs enter the ribosomal active site in the presence of a bound macrolide, they trigger distinct conformational rearrangements within the PTC, including reorientation of nucleotides A2062, U2585, U2506, and other residues of the 23S rRNA [11]. These structural changes disrupt the precise geometry required for catalysis of the peptide bond between the α-amino group of the aminoacyl-tRNA in the A site and the carbonyl group of the peptidyl-tRNA in the P site. As a result, even when both substrates are present, the transpeptidation reaction fails to occur, leading to ribosomal stalling and translational arrest [11,12]. Thus, macrolide-mediated inhibition of protein synthesis arises not from physical blockage of the tunnel but from allosteric modulation of PTC activity, driven by the interaction between the antibiotic and the nascent polypeptide chain [8,9]. This makes azithromycin a clear example of context-dependent translational inhibition, determined jointly by the structure of the drug and the sequence of the emerging peptide.

Azithromycin resistance in *N. gonorrhoeae* arises through several molecular mechanisms, including modification of the ribosomal target and enhanced drug efflux. In the *N. gonorrhoeae* genome, the *rrn* operon encoding rRNA is present in four copies, and macrolide susceptibility depends on the number of mutated alleles. The C2611T substitution in three or four alleles results in azithromycin minimum inhibitory concentration (MICs) of 2–16 mg/L [13], whereas the A2059G mutation in three or four alleles increases MIC to ≥256 mg/L, and up to 4096 mg/L when combined with MtrCDE overexpression, far exceeding the EUCAST and CLSI resistance breakpoint of 1 mg/L [14,15,16]. In contrast, isolates carrying A2059G or C2611T in only a single *rrn* copy exhibit MICs comparable to wild-type strains. However, under selective pressure in vitro, these mutations can rapidly spread to all four operons through homologous recombination [17].

A major contribution to azithromycin resistance is provided by the MtrCDE efflux system, whose activity can be modulated by alterations in both regulatory and coding regions of the corresponding operon. Several missense mutations in *mtrR*, including A39T, R44H, G45D/S, L47P, D79N, and M197I, reduce the DNA-binding activity of the MtrR repressor. In addition, deletions and substitutions within the promoter region (-35delA and -10insT/TT) disrupt *mtrR* expression [18,19]. Some clinical isolates also harbor frameshift mutations in *mtrC* and *mtrD* (e.g., deletions del349-GCGC-352 and del2736-GGGCCAATGC-2745), resulting in nonfunctional proteins and consequently increased susceptibility to macrolides [20,21]. Interestingly, mutations such as -35delA, A39T, and G45D in *mtrR*, which lead to overexpression of the MtrCDE efflux pump, reduce susceptibility not only to azithromycin but also to host-derived antimicrobial factors, including steroid hormones (e.g., progesterone) and the antimicrobial peptide LL-37, thereby enhancing bacterial survival in vivo in a murine infection model [22,23]. This dual advantage likely contributes to the high prevalence of these variants relative to other azithromycin resistance determinants.

Mosaic alleles of the *mtrCDE* operon, which arise through interspecies recombination among different *Neisseria* species, are of particular interest [24]. Among azithromycin-resistant *N. gonorrhoeae* isolates, these mosaic variants are now detected far more frequently than canonical resistance-associated mutations in the 23S rRNA gene [24,25,26]. Strong linkage disequilibrium and co-evolution have been observed between mosaic *mtrR* and *mtrD* alleles, as well as within the overlapping promoters of *mtrR* and *mtrCDE*, collectively driving overexpression of the MtrCDE efflux pump [24,25]. Furthermore, specific substitutions in MtrD, including R714H, S821A, and K823E/D, could alter the transporter’s structure and affect its binding to antimicrobials [18,19,27].

Mutations in ribosomal proteins that shape the nascent peptide exit tunnel also play an important role. A bacterial genome-wide association study (GWAS) identified the G70D substitution in ribosomal protein L4 (*rplD*) as significantly associated with elevated azithromycin MICs (*p* = 1.08 × 10^−11^), and introduction of this mutation into an isogenic background increased macrolide MICs by 3–6 fold without a measurable fitness cost in vitro [21]. Additional substitutions in the same macrolide-binding region (G68D, T69I, G70S), as well as tandem duplications, have been linked to even higher levels of resistance. Tandem duplications at the C-terminus of ribosomal protein L22 (*RplV*), at positions 83 (KGPSLK) and 90 (ARAK), confer moderate azithromycin resistance [28,29]. Experimental evolution studies further demonstrate that mutations in *RplV* (L22), *RplD* (L4), and *RpmH* (L34) frequently arise during early stages of azithromycin exposure and may serve as intermediate steps toward the development of high-level resistance [28].

Historically, azithromycin-resistant *N. gonorrhoeae* isolates carrying *erm* methyltransferases (ErmA, ErmB, ErmC, ErmF) have been reported [30]. These enzymes methylate A2058 in the 23S rRNA [31], and some isolates have also been found to harbor macrolide esterases (EreA, EreB) or the MefA efflux pump [32]. Although these determinants are widespread in other bacterial species, they are detected only rarely in *N. gonorrhoeae* and appear to play little, if any, role in the global landscape of macrolide resistance [26,33,34].

Taken together, azithromycin resistance in *N. gonorrhoeae* represents a polygenic trait shaped by the interplay of mutations in rRNA, ribosomal proteins forming the NPET, efflux systems, and their regulatory elements. The increasing prevalence of azithromycin-resistant isolates underscores the need to reassess the full spectrum of genetic determinants contributing to resistance and to understand their distribution within the population. To refine this spectrum, we performed a targeted association analysis of ribosomal genes, *rrn* operons, and key resistance loci, complemented by phylogenetic reconstruction to elucidate global relationships among isolates.

## 2. Results

### 2.1. Results of the Genome-Wide Association Study

The association analysis identified 113 polymorphisms significantly associated with reduced azithromycin susceptibility (FDR < 0.05) out of the 1293 variants examined. These resistance-associated markers included mutations within the ribosomal RNA operon (*rrl*, *rrs*, *rrf* genes, and intergenic regions), genes encoding proteins of the 50S ribosomal subunit (*rplA*, *rplB*, *rplC*, *rplD*, *rplE*, *rplF*, *rplM*, *rplN*, *rplO*, *rplT*, *rplW*, *rplX*, *rplY*, *rpmD*, *rpmJ*), as well as polymorphisms in the *porB* porin gene, the *mtrCDE* efflux pump, and its repressor *mtrR*. The strongest associations were observed for mutations in the 23S rRNA, homologous to A2059G (*rrl*_A2047G_4; β = 9.07) and C2611T (*rrl*_C2599T_4; β = 4.47), which remain key determinants of azithromycin resistance. Additional major contributors included variants in the *mtrCDE*/*mtrR* operon: frequent mutations in *mtrR* (-35 promoter deletion, meningitidis-like promoter, A39T, G45D; β = −0.74 to 1.68) and the recently described *mtrD* substitution K823E (β = 1.36). Among porin-associated markers, significant effects were observed for *porB1b*_G120K (β = 0.62) and *porB1b*_A121D/N (β = 0.38–0.50). We also identified multiple mutations in ribosomal proteins forming the NPET, including substitutions in L4 (*rplD*: G70D, A118T, R157Q, V125A, A147G; β = 0.44–2.14), L2 (*rplB*: D187N, S75P, V114A; β = 0.70–3.15), L3 (*rplC*_M151V; β = 0.37), and L23 (*rplW*: N38S, D90A, I74T, C28R; β = 0.49–1.87). Additional variants were detected in other 50S subunit proteins, including *rplT*_S75P (β = 4.15), *rplM*_G98D (β = 1.52), and *rplA*_T129I (β = 1.18). Notably, several rare but highly impactful substitutions were found in L5 (*rplE*_R59K/R64K; β = 4.28), L6 (*rplF*_K86R/S146A; β = 4.28), L14 (*rplN*_V47A; β = 4.28), L15 (*rplO*_V71A/D137A; β = 3.58–4.28), and L24 (*rplX*_G30D/K53E; β = 4.28), highlighting that extensive structural remodeling of the ribosome can markedly influence azithromycin binding.

In addition to these protein-coding variants, significant polymorphisms were detected in the 16S rRNA (*rrs*_C526T_1, β = 4.26; *rrs*_C1365T_1, β = 4.85) and in intergenic regions of the *rrn* operon (A1834G_2, β = 5.12; G1970GAGAAA_1, β = 1.19). Although these variants occur at low frequencies, they may exert substantial effects on resistance. Of particular interest is the protective effect of *mtrR*_A39T (β = −0.74), which is associated with increased azithromycin susceptibility. The most common polymorphisms (frequency ≥ 0.25%) are summarized in Table 1, and the complete list of all 113 significant variants is provided in Supplementary Table S1.

**Figure 1:**
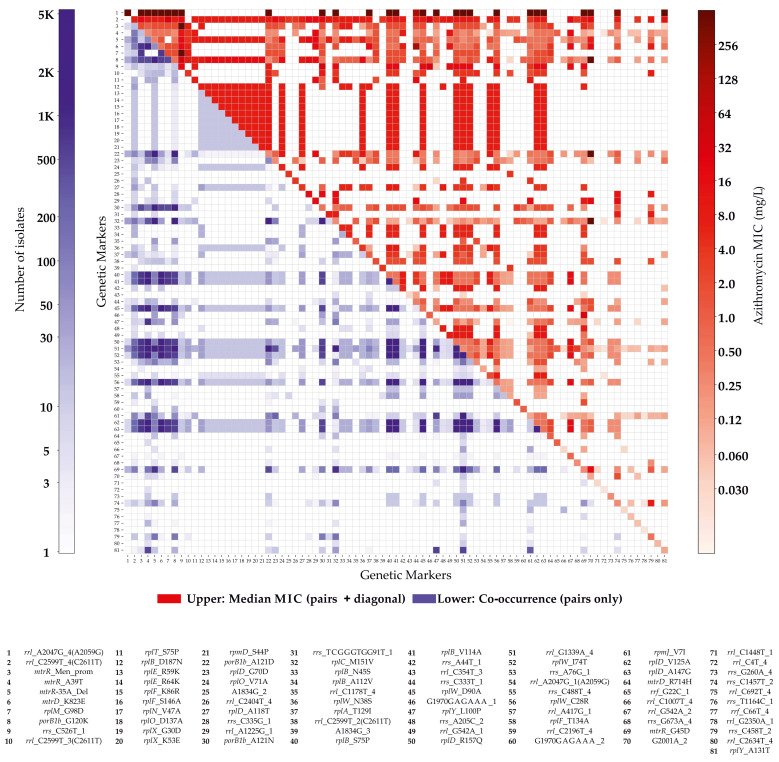
Distribution and phenotypic impact of azithromycin resistance markers. The lower heatmap illustrates the co-occurrence patterns of 81 most common genetic polymorphisms associated with azithromycin resistance. Rows and columns represent individual markers, and color intensity reflects the number of isolates carrying each specific combination of determinants; white cells indicate combinations not observed in the dataset. The upper heatmap shows the median azithromycin MIC values for isolates harboring the corresponding pairs of markers. Each cell represents the median MIC for isolates with a given marker combination, including direct comparison of the phenotypic effects of individual and combined mutations. Diagonal cells correspond to the median MIC values for isolates carrying the respective marker (regardless of the presence of other markers), whereas off-diagonal cells represent isolates carrying both markers. Darker colors correspond to higher MIC values, indicating increased levels of resistance.

To examine the distribution of azithromycin resistance markers across the global *N. gonorrhoeae* population and their contribution to MIC levels, we performed a comprehensive analysis of the identified genetic determinants. The prevalence of individual markers and their combinations, along with the median azithromycin MIC values associated with each pattern, is visualized in a heatmap (Figure 1). This representation highlights the frequency of co-occurring polymorphism combinations within the population and enables assessment of the phenotypic impact of individual mutations and their interactions on resistance.

Analysis of the co-occurrence patterns among the 113 significant genetic variants revealed well-defined linkage structures that reflect both the organization of ribosomal operons and the functional interdependence of individual resistance determinants. The densest clusters were observed within the ribosomal RNA operons and within the S10 ribosomal protein operon (including *rplB*, *rplC*, *rplD*, and *rplW*), where multiple polymorphisms form tightly linked variant clusters that are frequently co-inherited.

A particularly prominent cluster was located in the S10 operon, which includes proteins L2, L3, L4, L23, and components of the small subunit. Within this region, we observed both fully linked variants (e.g., *rplB* S75P with *rplB* A114V; *rplD* V125A with *rplD* A147G) and asymmetric linkage patterns in which one substitution consistently co-occurred with several others. This organization is consistent with the notion that alterations within the nascent peptide exit tunnel require coordinated compensatory changes to minimize functional costs.

Beyond intra-operon clusters, we also identified stable associations between mutations located in distinct ribosomal loci. For example, the L13 substitution *rplM* G98D frequently co-occurred with the NPET-associated cluster from the S10 operon, suggesting epistatic interactions between different structural domains of the ribosome.

To assess interactions between genetic determinants of resistance, we performed a systematic analysis of all possible pairwise combinations among the 113 significant markers. Of the 6328 theoretically possible pairs (113 × 112/2), 2080 were present in the dataset, and 341 occurred at sufficient frequency to permit statistical comparison (≥10 isolates in each of the “A only,” “B only,” and “A + B” groups). For each pair, the distribution of log_2_ (azithromycin MIC) values in the A + B group was compared with the corresponding single-mutation groups using a one-sided Mann–Whitney test followed by Benjamini–Hochberg correction (FDR < 0.05). A synergistic effect was defined as a statistically significant increase in MIC in the A + B group relative to both single-mutation groups. Conversely, an antagonistic effect was assigned when the MIC in the A + B group was significantly lower than in both single-mutation groups. All remaining combinations were classified as non-synergistic.

**Figure 2:**
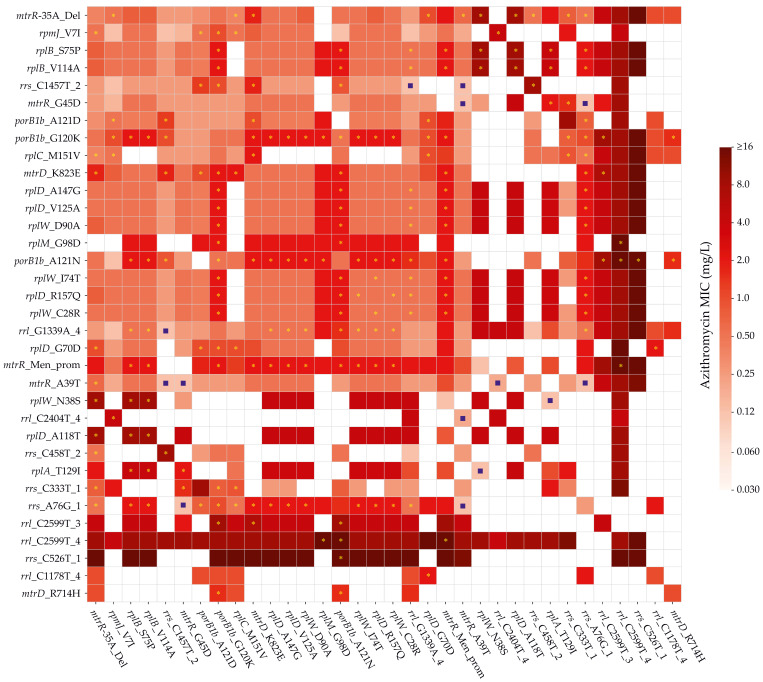
Heatmap of median azithromycin MIC values in *N. gonorrhoeae* for selected genetic markers. The heatmap shows the median MIC values for genetic markers represented by at least 10 isolates. Diagonal cells correspond to the median MIC values for isolates carrying the respective marker (regardless of the presence of other markers), whereas off-diagonal cells represent isolates carrying both markers simultaneously. The color scale is truncated: MIC values ≥ 16 mg/L are displayed using a single upperbound color, and the lower limit is fixed at 0.03 mg/L. White cells indicate marker combinations not observed in the dataset. Synergistic pairs are marked with yellow asterisks, whereas antagonistic pairs are indicated by blue squares.

This analysis identified 100 synergistic, 7 antagonistic, and 234 non-synergistic pairs. Quantitative evaluation showed that, for most synergistic combinations, the median MIC increased by one to two twofold dilution steps, consistent with predominantly additive or mildly supra-additive effects of combined mutations. However, several pairs exhibited pronounced synergy, resulting in 8- to 21-fold increases in median MIC (Figure 2 and Table 2). The strongest effects were observed for three types of combinations: (1) mutations in ribosomal proteins forming the NPET, e.g., rplB_S75P with rplD_A118T or rplW_N38S, resulting in a 16-fold increase; (2) mutations in distinct domains of the 16S rRNA (e.g., *rrs*_C1457T_2/*rrs*_C458T_2, 16-fold increase); and (3) regulatory alterations in the *mtr* locus combined with ribosomal protein mutations (e.g., *mtrR*_-35A_del with rplW_N38S, 21-fold increase). Notably, the strongest known determinant of azithromycin resistance such as *rrl*_A2047G (A2059G) did not participate in any synergistic interactions. A complete list of all tested pairs, including statistical significance and effect sizes, is provided in Supplementary Table S2.

### 2.2. Population Structure and Distribution of Resistance Lineages

**Figure 3:**
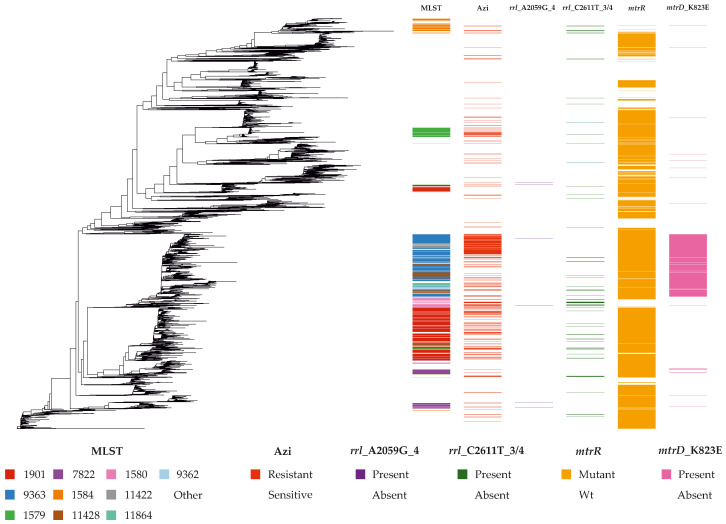
Maximum-likelihood phylogenetic tree based on the core-genome SNP alignment of 14,727 *Neisseria gonorrhoeae* isolates. The phylogeny is accompanied by a heatmap showing MLST types, azithromycin susceptibility phenotypes (MIC ≥ 1 mg/L), and resistance-associated genetic determinants, including *rrl*_A2047G_4 (A2059G), *rrl*_C2599T_3/4 (C2611T), polymorphisms in *mtrR*, and *mtrD*_K823E. The color scale indicates the presence (dark shading) or absence (light shading) of each variant, and column headers specify the corresponding mutations. For *mtrR*, the label “Mutant” denotes the presence of one or more mutations, including A39T, the meningitidis-like promoter, and the -35A deletion.

We assessed the relationships among 14,727 *N. gonorrhoeae* isolates using a SNP-based alignment of the core genome. To minimize the impact of homologous recombination on phylogenetic reconstruction, regions with anomalously high SNP density identified by Gubbins were removed. The resulting maximum-likelihood phylogeny is shown in Figure 3 and is accompanied by a heatmap illustrating MLST types, azithromycin susceptibility phenotypes, and the presence of key resistance determinants.

Analysis of azithromycin resistance (MIC ≥ 1 mg/L) across MLST types revealed that the resistant phenotype was not confined to a single clonal lineage but instead displayed a multi-focal, polyphyletic distribution. In the dataset as a whole, 15% of isolates were resistant, with a median azithromycin MIC of 0.25 mg/L. MLST types represented by at least 30 isolates and with ≥25% resistant isolates included: 11422, 8134, 9363, 1580, 9365, 7371, 1579, 8126, and 9362. One of the most common types globally, MLST type 1901, showed a resistance proportion of 23% (median MIC 0.5 mg/L). Below, we examine the characteristics of these types in greater detail.

Characterization of these lineages revealed three principal molecular strategies underlying resistance. Lineages ST-11422, ST-8134, and ST-9363 displayed a profile dominated by mutations in ribosomal proteins that form the nascent peptide exit tunnel (NPET), including *rplD* R157Q, V125A, and A147G (L4), as well as *rplW* D90A, I74T, and C28R (L23). Additional contributions came from mutations in proteins that stabilize the tunnel architecture, such as *rplB* S75P and V114A (L2), and *rplM* G98D (L13), which participates in subunit assembly and may influence ribosomal stability. These lineages also showed near-complete fixation of the structural variant *mtrD*_K823E (frequency >94%) and a high prevalence of regulatory mutations in the *mtrR* locus, including the meningitidis-like promoter variant (frequency 74–94%). In contrast, mutations in the 23S rRNA were exceedingly rare (≤2%).

Lineages ST-1580 and ST-8126 were characterized by a 23S-associated resistance profile, in which reduced susceptibility was primarily driven by *rrl* C2599T_4 and *rrl* A2047G_4 mutations in domain V of the 23S rRNA. These alterations frequently co-occurred with mutations in proteins that interact with the 23S rRNA and stabilize the peptidyl transferase center, including *rplM* G98D (L13) and *rplB* S75P/V114A (L2), as well as the G45D mutation in the repressor MtrR.

A third profile, associated with modulation of the influx-efflux balance, was represented by lineages ST-1901, ST-9365, ST-7371, and ST-1579. This strategy was marked by a high frequency of mutations in the PorB porin (G120K, A121D/N) and regulatory changes leading to overexpression of the MtrCDE efflux pump (e.g., the -35A promoter deletion in *mtrR* or the *mtrR* A39T substitution). Additional alterations included mutations in *rplC* M151V (L3, which contacts the PTC) and *rplM* G98D (L13), whereas NPET-associated markers were only partially represented in these lineages. Taken together, azithromycin resistance in the global *N. gonorrhoeae* population arises through at least three major molecular pathways: modification of the NPET, mutations in the 23S rRNA, and modulation of the influx-efflux balance. Each pathway is independently established in distinct evolutionary lineages and may be further complemented by additional genetic determinants. Detailed frequencies of key mutations across the examined MLST types are provided in Supplementary Table S3, and the associations between major MLST types and the most significant genetic variants are shown in Figure S1.

## 3. Discussion

### 3.1. Characterization of Identified Polymorphisms

The GWAS broadly confirmed the major determinants of azithromycin resistance in *N. gonorrhoeae* previously reported in the literature [28], while also expanding the range of implicated loci. As expected, high-level resistance was strongly associated with canonical mutations in domain V of the 23S rRNA (A2059G and C2611T in *E. coli* numbering, corresponding to A2047G and C2599T in *N. gonorrhoeae*). These variants remain the most penetrant markers of macrolide resistance. Likewise, alterations within the *mtr* locus (including promoter deletions in *mtrR*, coding sequence substitutions such as G45D, and mosaic *mtrD* alleles) were confirmed to significantly reduce susceptibility, consistent with the established role of the MtrCDE efflux pump in elevating azithromycin MICs.

Of particular importance is the confirmation of findings from [28] and [35] regarding the contribution of substitutions in ribosomal protein L4 (*rplD*), especially G70D, to elevated MICs. In our expanded dataset, *rplD*_G70D again showed a robust association with increased MICs, and additional substitutions within the macrolide-binding region of L4 (e.g., A118T) were identified. The relevance of previously described variants (V125A, A147G, and R157Q) was also reaffirmed [35]. These observations reinforce the concept that the NPET represents a recurrent target of adaptive remodeling under macrolide pressure. Although the magnitude of their effect is smaller than that of 23S rRNA mutations, the repeated independent emergence of these variants across diverse genetic backgrounds suggests that such polymorphisms may act as intermediate “stepping-stone” adaptations toward high-level resistance.

In addition to the key mutations in the 23S rRNA and ribosomal protein L4 (*rplD*), our genome-wide analysis identified several statistically significant associations involving other ribosomal proteins that form or interface with the NPET. The strongest effects were observed for rare but highly associated variants, including *rplB*_D187N (0.26%, β = 3.15), *rplD*_A118T (0.35%, β = 2.14), *rplM*_G98D (4.66%, β = 1.52), and *rplA*_T129I (0.62%, β = 1.18). These findings reinforce the notion that the NPET remains a major target of macrolide-driven selective pressure, and that rare variants with high β coefficients may represent critical amplification points in the development of the resistance phenotype. Alongside these rare but impactful substitutions, we also identified several high-frequency polymorphisms associated with moderate MIC shifts yet strong statistical significance. Variants such as *rplB*_S75P and *rplB*_V114A (both 17.62%, β = 0.70), *rplW*_D90A (17.71%, β = 0.63), *rplW*_I74T (17.79%, β = 0.51), *rplW*_C28R (17.80%, β = 0.49), *rplD*_R157Q (17.81%, β = 0.55), *rplD*_V125A and *rplD*_A147G (both 17.88%, β = 0.44) constitute a stable NPET-associated profile observed across multiple clonal lineages. Their high prevalence and moderate effect sizes suggest a potential role in stabilizing or reinforcing the resistance phenotype.

Additional significant associations were detected in genes encoding proteins located outside the NPET, including *rplY*_L100P (8.84%, β = −0.33), *rplY*_A131T (3.99%, β = −0.30), *rplF*_T134A (1.55%, β = −0.69), and *rpmJ*_V7I (13.18%, β = 0.27). Although the functional contribution of these proteins to macrolide resistance remains less well characterized, their statistical significance and substantial allele frequencies make them promising candidates for further investigation, particularly within the broader context of the polygenic architecture of azithromycin resistance.

Analysis of rRNA variation further confirmed the relevance of additional substitutions in both the 23S and 16S rRNA genes, including rrl_C2404T_4 (0.26%, β = 2.38), rrl_C1178T_4 (0.30%, β = 1.50), rrl_G1339A_4 (76.38%, β = 0.23), as well as rrs_C333T_1, rrs_A76G_1, rrs_C1457T_2, and rrs_C458T_2—all exhibiting FDR < 0.05 and β values ranging from 0.39 to 1.06. These findings suggest that macrolide-driven selective pressure may act not only on the canonical antibiotic-binding site but also on rRNA elements involved in translational dynamics, intersubunit coordination, and the initiation of protein synthesis. Beyond the canonical mechanisms, our analysis also identified statistically significant associations involving genetic variants whose functional contribution to macrolide resistance in *N. gonorrhoeae* remains to be fully clarified. These include amino acid G120K and A121D/N substitutions in the PorB porin. At first glance, the association between PorB mutations and elevated azithromycin MICs appears counterintuitive, as macrolides are traditionally viewed as compounds that traverse the outer membrane primarily through spontaneous diffusion across the lipid bilayer [36]. However, a synthesis of structural, biophysical, and computational evidence provides a coherent explanatory framework, extending principles established for hydrophilic molecules to the amphipathic azithromycin. The outer membrane of Gram-negative bacteria is a rigid, asymmetric structure in which β-barrel PorB proteins are embedded within a dense OMP-LPS-OMP network that shapes the local dielectric environment, hydration properties, and mobility of the surface layer [37,38,39]. Within this architecture, even single charge-altering substitutions in the L3 loop of PorB can influence not only the physicochemical properties of the pore itself but also the organization and dynamic state of the surrounding LPS matrix.

Time-resolved second harmonic light scattering experiments demonstrate that azithromycin interacts actively with the anionic surface of LPS and competes with cationic reporters for adsorption sites, with measurable changes in adsorption kinetics emerging only after sufficient accumulation of the antibiotic within the interfacial layer [40]. Studies using model membranes further show that azithromycin binds to lipid phosphate headgroups, reduces their mobility, and perturbs lateral membrane organization while leaving the hydrophobic core largely unaffected [41]. Together, these observations highlight that the LPS interfacial layer (its charge, dielectric properties, hydration, and packing density) constitutes a major component of the energetic barrier ΔG_influx_. These considerations allow a mechanistic interpretation of how PorB substitutions may reduce azithromycin influx. The G120K substitution increases the local positive potential at the pore vestibule, lowering the probability of initial adsorption of the cationic form of azithromycin onto the surrounding LPS and simultaneously reshaping the transverse electrostatic field within the constriction zone. In contrast, A121D acts through a different mechanism: it enhances local hydration and dielectric permittivity within the L3 region, stabilizing a structured water layer and increasing the energetic cost of transferring an amphipathic molecule through the constriction. As a result, ΔG_influx_ increases and the passive influx rate of azithromycin decreases. The neutral A121N substitution exerts a more moderate effect. It increases polarity and hydration within the L3 region but does not counterbalance the positive charge introduced by G120K, thereby amplifying its influence on the local electrostatic field. This is consistent with the observed cumulative epistasis between G120K and A121N. Collectively, these variants reduce azithromycin influx through both interfacial contributions (adsorption, LPS dielectric properties, water structuring) and intrapore effects (transverse electrostatic field, hydration barrier) to ΔG_influx_. Because the influx-efflux system is inherently nonlinear, even a moderate reduction in influx can lead to a pronounced decrease in periplasmic azithromycin concentration in the context of MtrCDE hyperexpression. This provides a mechanistic explanation for the robust synergy observed between *porB* variants and *mtrR*/*mtrD* alleles, as well as the compensatory epistasis of the G120K + A121D combination, in which opposite charges partially neutralize one another.

### 3.2. Epistatic Effects of Combined Polymorphisms

The pairwise interaction analysis underscores the inherently complex and multilayered nature of azithromycin resistance in *N. gonorrhoeae*. Rather than arising from isolated mutations, resistance emerges through the coordinated action of multiple genetic determinants whose combined effects exceed the sum of their individual contributions. The observed distribution of interaction types (100 synergistic, 234 non-synergistic, and 7 antagonistic pairs) highlights the presence of a structured epistatic landscape. This pattern supports an integrative model in which azithromycin MICs are shaped by the interplay of several functionally distinct components. Central to this framework is the activity of the MtrCDE efflux system, which modulates intracellular drug concentration. Equally important is the porin-mediated permeability of the outer membrane, where amino acid substitutions in PorB alter the local electrostatic environment at the pore vestibule and thereby influence the energetics of antibiotic influx. A third major contributor is the ribosomal affinity for macrolides, determined by the mutational state of the 23S rRNA and associated ribosomal proteins that define the architecture of the nascent peptide exit tunnel. Together, these components form a cohesive mechanistic network in which epistatic interactions play a decisive role in shaping the resistance phenotype.

The structure of synergistic interactions reveals a clear functional logic. The most frequent synergistic combinations involve mutations that act at different levels of intracellular antibiotic control. Enhanced efflux (e.g., *mtrR*_-35A_del, *mtrR*_G45D, *mtrD*_K823E) commonly co-occurs with alterations in ribosomal proteins (*rplB*_S75P, *rplB*_V114A, *rplW*_N38S, *rplD*_A118T, *rplD*_V125A, *rplD*_A147G) or in the 23S/16S rRNA (*rrl*_C2599T, *rrs*_A76G, *rrs*_C1457T). These combinations constitute a classical “two-tier” mechanism in which one allele reduces intracellular accumulation of azithromycin, while the other decreases its affinity for the ribosome. Such pairs produce the most pronounced cumulative effects, fully consistent with a model of functional complementarity.

A particularly illustrative subset of synergistic interactions involves PorB. The G120K and A121N substitutions frequently co-occur with ribosomal mutations (*rplB*_S75P, *rplB*_V114A, *rplW*_C28R, *rplW*_D90A, *rplD*_V125A, *rplD*_A147G, *rplD*_R157Q) as well as with mutations in the 23S/16S rRNA (*rrl*_C2599T, *rrs*_A76G, *rrs*_C1457T). These combinations consistently exhibit strong synergy, in line with the fact that G120K and A121N reduce porin-mediated influx and thereby amplify the effects of mutations that diminish macrolide affinity for the ribosome. Notably, A121N is a neutral substitution that does not counterbalance the positive charge introduced by G120K, allowing both mutations within the same L3 loop to act cumulatively and intensify the electrostatic and structural distortion of the eyelet region.

Interactions within PorB also reveal more complex scenarios. The G120K + A121D combination is not among the synergistic pairs and is statistically associated with lower MIC values than G120K alone, while still conferring higher resistance than A121D. Our bidirectional analysis indicates that G120K + A121D functions as a partial “attenuator” of the G120K effect. This observation aligns with the charge-based model: the positively charged lysine at position 120 and the negatively charged aspartate at position 121 partially neutralize one another, reducing the local positive potential within the constriction zone and decreasing ion selectivity. Thus, PorB exhibits both additive (G120K + A121N) and compensatory (G120K + A121D) forms of epistasis, highlighting the sensitivity of the L3 loop’s electrostatic landscape to single-residue perturbations.

In contrast, the absence of synergy or epistasis is characteristic of combinations that act on the same limiting component of the resistance pathway. For example, different mutations within the regulatory *mtrR* locus (such as *mtrR*_G45D together with *mtrR*_-35A_del) do not produce an additive effect, because the stronger allele already achieves maximal derepression of MtrCDE, leaving no further capacity for enhancement by the second mutation. Similarly, multiple alterations within a single functional domain of the ribosome, such as domain V of the 23S rRNA or within an individual ribosomal protein, often exert overlapping effects that lead to phenotypic saturation.

Taken together, the interaction landscape shows that peak resistance occurs when mutations concurrently diminish azithromycin accumulation (through MtrCDE-mediated efflux and porin-dependent influx) and reduce its ribosomal affinity. Intrapore interactions within PorB constitute a distinct class of epistatic effects, capable of either amplifying or attenuating one another depending on the charge properties of the substitutions involved. These patterns reinforce the proposed model of functional complementarity and highlight the importance of accounting for epistasis when predicting MIC values from genomic data or assessing the evolutionary potential of circulating lineages.

This conceptual framework is directly reflected in the strategies employed by successful epidemiological lineages. The clonal complexes identified in our analysis exhibit diverse yet mechanistically coherent combinations of determinants. Lineages with NPET-dominated profiles (e.g., ST-11422, ST-8134) combine a fixed set of ribosomal mutations with universal activation of MtrCDE (*mtrD*_K823E), forming a strong synergistic barrier in the absence of canonical 23S rRNA mutations. Lineages with mixed profiles (e.g., ST-1580, ST-7371) sequentially integrate strong efflux, PorB alterations, and ribosomal mutations, engaging multiple mechanisms simultaneously.

Overall, our findings underscore that azithromycin resistance in *N. gonorrhoeae* is shaped by functional complementarity among independent mechanisms.

The most effective combinations concurrently reduce intracellular drug accumulation (via direct MtrCDE efflux and indirect PorB-mediated modulation of membrane electrostatics) and alter the antibiotic’s target. Understanding these interaction rules is essential for accurate genomic prediction of resistance levels and for evaluating the evolutionary trajectories of circulating genetic lineages.

### 3.3. Evolutionary Success of Azithromycin-Resistant Lineages

The global molecular epidemiological analysis revealed several genetic lineages of *N. gonorrhoeae* distinguished by distinct combinations of azithromycin resistance mechanisms. Nine MLST types (ST-11422, ST-8134, ST-9363, ST-1580, ST-9365, ST-7371, ST-1579, ST-8126, and ST-9362) were identified as particularly relevant, each represented by at least 30 isolates and exhibiting a proportion of resistant strains exceeding 25%. In addition, the widely distributed lineage ST-1901 (*n* = 1825) displayed a resistance rate of 23%, making it a substantial reservoir of azithromycin resistance in the global population.

The first group comprises lineages dominated by polymorphisms in ribosomal proteins. ST-11422 and ST-8134 show near-complete fixation of mutations affecting proteins that form the NPET, including *rplB* S75P and V114A, *rplD* V125A, A147G, and R157Q, and *rplW* C28R, I74T, and D90A. These alterations co-occur with the mutation *mtrD* K823E and a high prevalence of *rplM* G98D (>70%), while canonical 23S rRNA mutations (A2047G, C2599T) are virtually absent. This profile reflects a strategy in which high-level resistance is achieved primarily through the cooperative action of multiple NPET substitutions combined with enhanced efflux, consistent with the principle of functional complementarity.

The second group consists of mixed profiles dominated by 23S rRNA mutations. In ST-1580 and ST-8126, alterations in the 23S rRNA play a central role. The *rrl* C2599T_4 variant is present in 62.7% of resistant isolates in ST-1580 and in 90.9% of those in ST-8126. These mutations co-occur with high frequencies of *mtrR* G45D (89.8% and 31.8%, respectively) and a fixed NPET mutation set. This configuration suggests synergy between reduced target affinity, enhanced efflux, and ribosomal remodeling, enabling high resistance levels without apparent epidemiological fitness penalties.

The third group comprises porin–ribosomal lineages. ST-9365, ST-7371, and ST-1579 are characterized by high frequencies of PorB mutations (G120K, A121D/N) and the ribosomal protein L3 substitution *rplC* M151V, together with near-universal regulatory changes leading to MtrCDE hyperexpression (e.g., *mtrR* -35A_del). In these lineages, 23S rRNA mutations are less common or moderately represented, and the canonical NPET mutation set is absent. This pattern indicates an alternative evolutionary trajectory in which membrane permeability and efflux play dominant roles, complemented by moderate ribosomal alterations.

A fourth category includes hybrid profiles. ST-9363 occupies an intermediate position, combining near-complete fixation of NPET mutations (*rplB* S75P/V114A, *rplD* R157Q, *rplW* C28R, I74T, D90A) with a very high prevalence of *mtrD* K823E (99.4%) and moderate representation of 23S rRNA mutations (C2599T in 14.2% of resistant isolates). This profile illustrates the lineage’s flexibility and its capacity to adapt to diverse selective pressures.

Finally, ST-1901 represents a special case. Despite a relatively moderate proportion of resistant isolates (23%), its large population size makes it an important epidemiological reservoir. Its profile aligns with the porin–ribosomal category: high frequencies of *porB* G120K/A121N and *rplC* M151V, together with a notable proportion of 23S rRNA mutations (A2047G, C2599T), indicate a hybrid strategy that confers resistance while preserving the potential for further escalation.

Overall, azithromycin resistance in the global *N. gonorrhoeae* population does not stem from the expansion of a single highly resistant clone. Instead, it is sustained by several prevalent genetic lineages, each deploying a unique suite of mechanisms, including NPET-dominated, mixed, and porin-regulatory strategies. These lineages represent priority targets for enhanced epidemiological surveillance, as their continued spread or acquisition of additional determinants may lead to new outbreaks of resistant infections. The robustness of these findings is supported by the global scope of the dataset, comprising more than 14,700 genomes from over 60 countries across all continents.

### 3.4. Limitations

Several limitations of this study should be considered when interpreting these findings. First, the analysis relies on publicly available genomic data and metadata obtained from international repositories. Although these databases capture a broad spectrum of *N. gonorrhoeae* diversity, they may be subject to sampling biases, including overrepresentation of isolates from specific regions, time periods, or surveillance programs. Nevertheless, the large size of the dataset and its extensive geographic coverage suggest that such biases are unlikely to substantially affect the overall conclusions.

Second, azithromycin MICs were determined using different methodologies across laboratories, with variation in media, inoculum preparation, and interpretive criteria. This heterogeneity may introduce additional noise into quantitative associations between genetic variants and resistance phenotypes.

Third, although the GWAS identified numerous variants statistically associated with reduced susceptibility, the functional significance of many rare mutations remains uncertain. Experimental validation, such as allelic replacement or phenotypic characterization of isogenic strains, is required to confirm their causal contribution to resistance.

Finally, our analysis encompassed all *rrn* genes, large-subunit ribosomal proteins, and the full range of antimicrobial resistance determinants described in the literature. However, we acknowledge that additional mechanisms, such as epigenetic regulation, post-transcriptional modifications, or compensatory mutations outside canonical loci, may also influence the resistance phenotype and fall beyond the scope of this study.

## 4. Materials and Methods

### 4.1. Genome Collection and Curation

Whole-genome assemblies and associated metadata of *N. gonorrhoeae*, including azithromycin MICs, were retrieved from Pathogenwatch [42] (https://pathogen.watch/) and PubMLST [43] (https://pubmlst.org/) databases accessed on 1 May 2025. Duplicate assemblies were removed, and only isolates with available raw sequencing reads in the Sequence Read Archive (SRA) and documented azithromycin MICs were retained. The final dataset comprised 14,727 unique genomes (Supplementary Table S4). The collection represents isolates from all continents and 71 countries or territories. In Africa, 13 countries contributed a total of 214 isolates. Europe was most strongly represented, with 31 countries and 6864 isolates. Asia included 15 countries with 828 isolates, while North America accounted for 4968 isolates from five countries and territories, primarily the United States and Canada. Latin America contributed 808 isolates from four countries. Oceania was represented by Australia and New Zealand with 1043 isolates. In addition, two isolates had no specified country of origin. Altogether, this broad geographic distribution underscores the global representativeness of the dataset and provides a robust foundation for molecular epidemiological analysis of azithromycin resistance in *N. gonorrhoeae*. In total, 12,519 isolates exhibited MIC < 1 mg/L, while 2208 isolates had MIC ≥ 1 mg/L. In accordance with current EUCAST recommendations, azithromycin is used clinically only in combination with another effective agent; however, for surveillance and detection of acquired resistance mechanisms, the epidemiological cutoff value (ECOFF) of 1 mg/L was applied to classify isolates.

### 4.2. Identification of Known Antimicrobial Resistance Determinants

Known resistance-conferring mutations and acquired genes were identified using NCBI AMRFinderPlus v4.0.23 with the 2023 database and the --plus option to include resistance-associated point mutations [44]. Significant polymorphisms in *mtrR* and its promoter region were identified using BLASTn (default parameters, e-value 1 × 10^−10^) according to the NG-STAR scheme. Amino acid substitutions at positions 714 and 823 in *mtrD* were identified using BLASTn v2.16.0 with default parameters and an e-value threshold of 1 × 10^−10^.

### 4.3. Quantification of 23S rRNA Gene Mutations

A custom Snakemake v9.8.1 workflow was used to quantify mutations in the multicopy *rrn* operon (available at https://github.com/KostyaTheKing/rrn_pipeline, accessed on 1 May 2025). Raw reads were mapped with BWA-MEM v0.7.19-r1273 to a modified reference genome of a strain WHO_F_2024 (GCA_040383235.1) in which three of the four *rrn* operons were removed, leaving a single operon on the “+” strand. Variants in the target region were called using FreeBayes v1.3.5 with --ploidy 4. Allele copy number (0–4) was estimated by rounding the proportion of supporting reads to the nearest quartile.

### 4.4. Ribosomal Protein Mutations

Allelic variants of ribosomal protein genes were determined using mlst v2.23.0. Protein sequence alignments were generated with MAFFT v7.475 [45]. Positions with non-conservative substitutions in >0.1% of isolates were considered for downstream analyses.

### 4.5. Core-Genome Alignment and Distance Matrix

Core-genome MLST (cgMLST v2.0) profiles were generated using the mlst v2.23.0 (https://github.com/tseemann/mlst, accessed on 1 May 2025); missing loci were resolved by BLAST (v2.16.0) [46] against the scheme’s allele database. Allele sequences were aligned individually with MAFFT v7.475 (--auto) and concatenated. Missing alleles were replaced with N’s of corresponding length. Pairwise SNP distances were calculated from the core-genome alignment using snp-dists v0.8.2 and converted to a kinship matrix for GWAS.

### 4.6. Genome-Wide Association Study

Associations between genetic variants and azithromycin log_2_ (MIC) were tested using pyseer v1.3.9 with a linear mixed model and the kinship matrix to account for population structure [47]. For each variant, both *p*-values and regression coefficients (β) were estimated, quantifying the magnitude and direction of the effect. Because the dependent variable was log_2_-transformed, β-coefficients were interpreted as the change in log_2_ (MIC) associated with the presence of a given variant; accordingly, an increase in β by 1 corresponds to a two-fold increase in MIC, whereas β = 2 indicates an approximately four-fold elevation of MIC in linear scale. Multiple testing correction was performed using the Benjamini–Hochberg procedure (FDR < 0.05).

### 4.7. Assessment of Synergistic Interactions Between Resistance Markers

For each pair of resistance markers, isolates were partitioned into three mutually exclusive groups: those carrying marker A only, marker B only, or both markers (A + B). Azithromycin MIC values were log_2_-transformed, and the distribution within each group was summarized by the median. Interaction effects were assessed by comparing MIC values in the A + B group with each single-marker group using one-sided Mann–Whitney U tests, testing for shifts toward higher or lower MIC values. Raw *p*-values from the Mann–Whitney tests were subsequently adjusted using the Benjamini–Hochberg procedure, and statistical significance was defined as FDR < 0.05. A synergistic effect was defined as a statistically significant increase in MIC in the A + B group relative to both single-marker groups. An antagonistic effect was defined as a statistically significant decrease in MIC in the A + B group relative to both comparators. All other combinations were classified as non-synergistic. Only marker pairs represented by at least 10 isolates in each of the three groups were included in the analysis.

### 4.8. Phylogenetic Reconstruction

Recombinant regions were removed from the core-genome alignment using Gubbins v3.4.3. A maximum-likelihood phylogeny was inferred from the recombination-filtered alignment using IQ-TREE 2 v2.4.0 with the GTR + ASC model with ascertainment bias correction [48]. The best-scoring tree was selected from ten independent searches, and branch support was assessed using 1000 replicates of the SH-like approximate likelihood ratio test (SH-aLRT) and approximate Bayes tests [49,50]. All IQ-TREE analyses were performed with a fixed random seed (38) for reproducibility. Tree visualization and annotation were performed in R [51] using the packages ggtree [52], ggplot2, ggnewscale, dplyr, gtable, patchwork and some custom scripts.

## 5. Conclusions

Our large-scale GWAS of *N. gonorrhoeae* identified 113 genetic variants linked to azithromycin resistance, expanding the known resistome to include mutations in 16 ribosomal proteins, the efflux variant MtrD K823E, and PorB alterations. We show that resistance is polygenic and driven by functional synergy between mechanisms that reduce intracellular drug accumulation (efflux, membrane permeability) and those that alter the ribosome target. Three distinct molecular strategies have evolved convergently across successful global lineages: NPET-dominated, 23S rRNA-associated, and porin/efflux-mediated. These findings highlight the need to consider genetic interactions for accurate resistance prediction and surveillance, pointing to high-risk clones that threaten current therapy.

## Figures and Tables

**Table 1:** Key genetic variants associated with increased azithromycin MICs in *N. gonorrhoeae*. The table includes only variants present in the population at a frequency of ≥0.25% (*n* ≥ 37 isolates) and with FDR < 0.05. β represents the regression coefficient describing the contribution of each polymorphism to log_2_ (azithromycin MIC) in the linear mixed model. Because *N. gonorrhoeae* carries four copies of the ribosomal RNA operon (*rrn*), mutations in *rrl* (23S rRNA), *rrs* (16S rRNA), *rrf* (5S rRNA), and intergenic regions are annotated with a suffix indicating the number of operons in which the mutation was detected (e.g., *rrl*_A2047G_4 (A2059G) denotes the presence of the mutation in all four copies).

Genetic Variant	Frequency (%)	Adjusted *p* Value (FDR)	Effect Size (β)	Functional Annotation
*rrl*_A2047G_4 (A2059G)	0.34%	0	9.07	Homologous to A2059G in *E. coli* 23S rRNA (*rrl*)
*rrl*_C2599T_4 (C2611T)	3.18%	0	4.47	Homologous to C2611T in *E. coli* 23S rRNA (*rrl*)
*mtrR*_Meningitidis-like promoter	5.44%	1.79 × 10^−71^	1.68	*mtrR* promoter region (regulatory element of efflux pump)
*mtrR*_A39T	37.93%	4.85 × 10^−65^	−0.74	*mtrR* (repressor of the MtrCDE efflux pump)
*mtrR*_-35A Del	33.83%	7.68 × 10^−54^	0.88	*mtrR* promoter region (regulatory element of efflux pump)
*mtrD*_K823E	15.88%	8.99 × 10^−47^	1.36	*mtrD* (inner membrane transporter of the MtrCDE RND efflux pump)
*rplM*_G98D	4.66%	2.83 × 10^−46^	1.52	Ribosomal protein L13 (*rplM*)
*porB1b* _G120K	28.30%	6.71 × 10^−44^	0.62	Porin channel protein (*porB1b*)
*rrl*_C2599T_3 (C2611T)	0.37%	6.03 × 10^−37^	2.75	Homologous to C2611T in *E. coli* 23S rRNA (*rrl*)
*rplB*_D187N	0.26%	2.88 × 10^−28^	3.15	Ribosomal protein L2 (*rplB*)
*porB1b* _A121D	15.85%	1.06 × 10^−20^	0.50	Porin channel protein (*porB1b*)
*rplD*_G70D	2.94%	4.14 × 10^−20^	0.91	Ribosomal protein L4 (*rplD*)
*rrl*_C2404T_4	0.26%	7.76 × 10^−18^	2.38	Homologous to C2416T in *E. coli* 23S rRNA (*rrl*)
*rplD*_A118T	0.35%	8.28 × 10^−17^	2.14	Ribosomal protein L4 (*rplD*)
*porB1b* _A121N	12.98%	5.17 × 10^−9^	0.38	Porin channel protein (*porB1b*)
*rplC*_M151V	32.25%	1.04 × 10^−8^	0.37	Ribosomal protein L3 (*rplC*)
*rrl*_C1178T_4	0.30%	3.27 × 10^−8^	1.50	Homologous to T1180 in *E. coli* 23S rRNA (*rrl*)
*rplA*_T129I	0.62%	2.66 × 10^−7^	1.18	Ribosomal protein L1 (*rplA*)
*rrl*_C2599T_2 (C2611T)	0.25%	3.19 × 10^−7^	1.47	Homologous to C2611T in *E. coli* 23S rRNA (*rrl*)
*rplB*_S75P	17.62%	8.10 × 10^−7^	0.70	Ribosomal protein L2 (*rplB*)
*rplB*_V114A	17.62%	8.10 × 10^−7^	0.70	Ribosomal protein L2 (*rplB*)
*rrs*_C333T_1	0.40%	5.92 × 10^−6^	1.06	Homologous to T333 in *E. coli* 16S rRNA (*rrs*)
*rplW*_D90A	17.71%	1.33 × 10^−5^	0.63	Ribosomal protein L23 (*rplW*)
G1970GAGAAA_1	0.28%	4.86 × 10^−5^	1.19	Intergenic region of the *rrn* operon (position relative to the start of *rrs*)
*rplY*_L100P	8.84%	5.17 × 10^−5^	−0.33	Ribosomal protein L25 (*rplY*)
*rplD*_R157Q	17.81%	2.72 × 10^−4^	0.55	Ribosomal protein L4 (*rplD*)
*rrl*_G1339A_4	76.38%	5.49 × 10^−4^	0.23	Homologous to G1341A in *E. coli* 23S rRNA (*rrl*)
*rplW*_I74T	17.79%	6.75 × 10^−4^	0.51	Ribosomal protein L23 (*rplW*)
*rrs*_A76G 1	1.27%	8.06 × 10^−4^	0.49	Homologous to G76 in *E. coli* 16S rRNA (*rrs*)
*rplW*_C28R	17.80%	1.32 × 10^−3^	0.49	Ribosomal protein L23 (*rplW*)
*rplF*_T134A	1.55%	2.16 × 10^−3^	−0.69	Ribosomal protein L6 (*rplF*)
*rpmJ*_V7I	13.18%	3.49 × 10^−3^	0.27	Ribosomal protein L36 (*rpmJ*)
*rplD*_V125A	17.88%	5.44 × 10^−3^	0.44	Ribosomal protein L4 (*rplD*)
*rplD*_A147G	17.88%	5.44 × 10^−3^	0.44	Ribosomal protein L4 (*rplD*)
*mtrR*_G45D	12.30%	1.43 × 10^−2^	0.19	*mtrR* (repressor of the MtrCDE efflux pump)

**Table 2:** Representative * synergistic interactions between genetic determinants that increase azithromycin MIC in *N. gonorrhoeae*.

Marker A	Marker B	Median MIC (A Without B)	Median MIC (B Without A)	Median MIC (A + B)	Fold-Change
*mtrR*_Meningitidis-like promoter	*rrl*_C2599T_4 (C2611T)	2.00	8.00	16.00	2.0
*mtrR*_Meningitidis-like promoter	*porB1b*_G120K	1.22	0.38	2.00	1.6
*mtrR*_Meningitidis-like promoter	*porB1b*_A121N	1.00	0.50	2.00	2.0
*mtrR*_Meningitidis-like promoter	*rplB*_S75P	1.00	0.50	2.00	2.0
*mtrR*_Meningitidis-like promoter	*rplB*_V114A	1.00	0.50	2.00	2.0
*mtrR*_Meningitidis-like promoter	*rplW*_D90A	1.00	0.50	2.00	2.0
*mtrR*_Meningitidis-like promoter	*rplD*_R157Q	1.00	0.50	2.00	2.0
*mtrR*_Meningitidis-like promoter	*rplW*_I74T	1.00	0.50	2.00	2.0
*mtrR*_Meningitidis-like promoter	*rplW*_C28R	1.00	0.50	2.00	2.0
*mtrR*_Meningitidis-like promoter	*rplD*_A147G	1.00	0.50	2.00	2.0
*mtrR*_Meningitidis-like promoter	*rplD*_V125A	1.00	0.50	2.00	2.0
*mtrR*_-35A Del	*rplD*_A118T	0.38	3.46	8.00	2.3
*mtrR*_-35A Del	*rplW*_N38S	0.38	0.13	8.00	21.1
*mtrD*_K823E	*mtrR*_Meningitidis-like promoter	0.50	0.38	2.00	4.0
*mtrD*_K823E	*porB1b*_G120K	0.50	0.38	2.00	4.0
*mtrD*_K823E	*rrl*_C2599T_3 (C2611T)	0.50	2.00	6.00	3.0
*mtrD*_K823E	*porB1b*_A121N	0.50	0.50	2.00	4.0
*mtrD*_K823E	*rplC*_M151V	0.50	0.25	2.00	4.0
*mtrD*_K823E	*rrs*_A76G_1	0.50	0.25	2.00	4.0
*rplM*_G98D	*rrl*_C2599T_4 (C2611T)	2.00	8.00	32.00	4.0
*porB1b*_G120K	*rplM*_G98D	0.38	1.00	2.00	2.0
*porB1b*_G120K	*rrl*_C2599T_3 (C2611T)	0.50	2.00	8.00	4.0
*porB1b*_G120K	*rplB*_S75P	0.38	0.50	2.00	4.0
*porB1b*_G120K	*rplB*_V114A	0.38	0.50	2.00	4.0
*porB1b*_G120K	*rplW*_D90A	0.38	0.50	2.00	4.0
*porB1b*_G120K	*rplD*_R157Q	0.38	0.50	2.00	4.0
*porB1b*_G120K	*rplW*_I74T	0.38	0.50	2.00	4.0
*porB1b*_G120K	*rplW*_C28R	0.38	0.50	2.00	4.0
*porB1b*_G120K	*rplD*_A147G	0.38	0.50	2.00	4.0
*porB1b*_G120K	*rplD*_V125A	0.38	0.50	2.00	4.0
*porB1b*_A121N	*rrl*_C2599T_4 (C2611T)	0.50	8.00	16.00	2.0
*porB1b*_A121N	*rrl*_C2599T_3 (C2611T)	0.50	4.00	8.00	2.0
*porB1b*_A121N	*rplM*_G98D	0.50	1.00	2.00	2.0
*porB1b*_A121N	*rrs*_C526T_1	0.50	8.00	32.00	4.0
*porB1b*_A121N	*rplB*_S75P	0.50	0.50	2.00	4.0
*porB1b*_A121N	*rplB*_V114A	0.50	0.50	2.00	4.0
*porB1b*_A121N	*rplW*_D90A	0.50	0.50	2.00	4.0
*porB1b*_A121N	*rplD*_R157Q	0.50	0.50	2.00	4.0
*porB1b*_A121N	*rplW*_I74T	0.50	0.50	2.00	4.0
*porB1b*_A121N	*rplW*_C28R	0.50	0.50	2.00	4.0
*porB1b*_A121N	*rplD*_A147G	0.50	0.50	2.00	4.0
*porB1b*_A121N	*rplD*_V125A	0.50	0.50	2.00	4.0
*rplA*_T129I	*rplB*_S75P	0.38	0.50	4.00	8.0
*rplA*_T129I	*rplB*_V114A	0.38	0.50	4.00	8.0
*rplB*_S75P	*rplD*_A118T	0.50	0.13	8.00	16.0
*rplB*_S75P	*rplW*_N38S	0.50	0.13	8.00	16.0
*rplB*_S75P	*rrs*_A76G_1	0.50	0.25	2.00	4.0
*rplB*_V114A	*rplD*_A118T	0.50	0.13	8.00	16.0
*rplB*_V114A	*rplW*_N38S	0.50	0.13	8.00	16.0
*rplB*_V114A	*rrs*_A76G_1	0.50	0.25	2.00	4.0
*rplW*_D90A	*rrs*_A76G_1	0.50	0.25	2.00	4.0
*rplD*_R157Q	*rrs*_A76G_1	0.50	0.25	2.00	4.0
*rplW*_I74T	*rrs*_A76G_1	0.50	0.25	2.00	4.0
*rplW*_C28R	*rrs*_A76G_1	0.50	0.25	2.00	4.0
*rpmJ*_V7I	*rrl*_C2404T_4	0.13	0.19	4.00	21.1
*rplD*_A147G	*rrs*_A76G_1	0.50	0.25	2.00	4.0
*rplD*_V125A	*rrs*_A76G_1	0.50	0.25	2.00	4.0
*mtrR*_G45D	*rplA*_T129I	0.25	0.13	2.00	8.0
*rrs*_C1457T_2	*rrs*_C458T_2	0.25	0.50	8.00	16.0

* Note: The table lists marker pairs demonstrating a statistically significant synergistic effect (one-sided Mann–Whitney U test with Benjamini–Hochberg correction, FDR < 0.05) for which the median azithromycin MIC in the combined-mutation group (A + B) was ≥2 mg/L. Synergy was defined as a significant shift in the log_2_ (MIC) distribution in the A + B group toward higher values compared with each corresponding single-mutation group (“A only” and “B only”), with all groups containing at least 10 isolates. Fold-change was calculated as the ratio of the median MIC in the A + B group to the highest median MIC observed among the two single-mutation groups. A complete list of all tested pairs is provided in Supplementary Table S2.

## Data Availability

All data supporting the findings of this study are provided within the article and its Supplementary Materials. Supplementary Tables S1–S4 and Supplementary Figure S1 contain the complete datasets, GWAS results, synergy analyses, phylogenetic reconstruction, and associated metadata. Additional information can be obtained from the corresponding author upon reasonable request.

## Supplementary Materials

The following supporting information can be downloaded at: https://www.mdpi.com/article/10.3390/ijms27052258/s1.

## Abbreviations

The following abbreviations are used in this manuscript:

AZI	Azithromycin
CLSI	Clinical and Laboratory Standards Institute
cgMLST	core-genome Multilocus Sequence Typing
ECOFF	Epidemiological cutoff value
EUCAST	European Committee on Antimicrobial Susceptibility Testing
FDR	False Discovery Rate
GWAS	Genome-Wide Association Study
LPS	Lipopolysaccharide
MAMs	Macrolide-Arrest Motifs
MIC	Minimum Inhibitory Concentration
MLST	Multilocus Sequence Typing
NPET	Nascent Peptide Exit Tunnel
OMP	Outer Membrane Protein
PTC	Peptidyl Transferase Center
RND	Resistance-Nodulation-Division (efflux pump family)
*rrn*	Ribosomal RNA Operon
SNP	Single-Nucleotide Polymorphism
SRA	Sequence Read Archive
WHO	World Health Organization
