# Supplementary figures and images for: The Synergistic Armory: A Global Genome-Wide Association Study Reveals the Integrated Mechanisms of Azithromycin Resistance in Neisseria gonorrhoeae
Source: Int J Mol Sci. 2026 Feb 27;27(5):2258. doi: 10.3390/ijms27052258 (PMC12984986; doi:10.3390/ijms27052258)

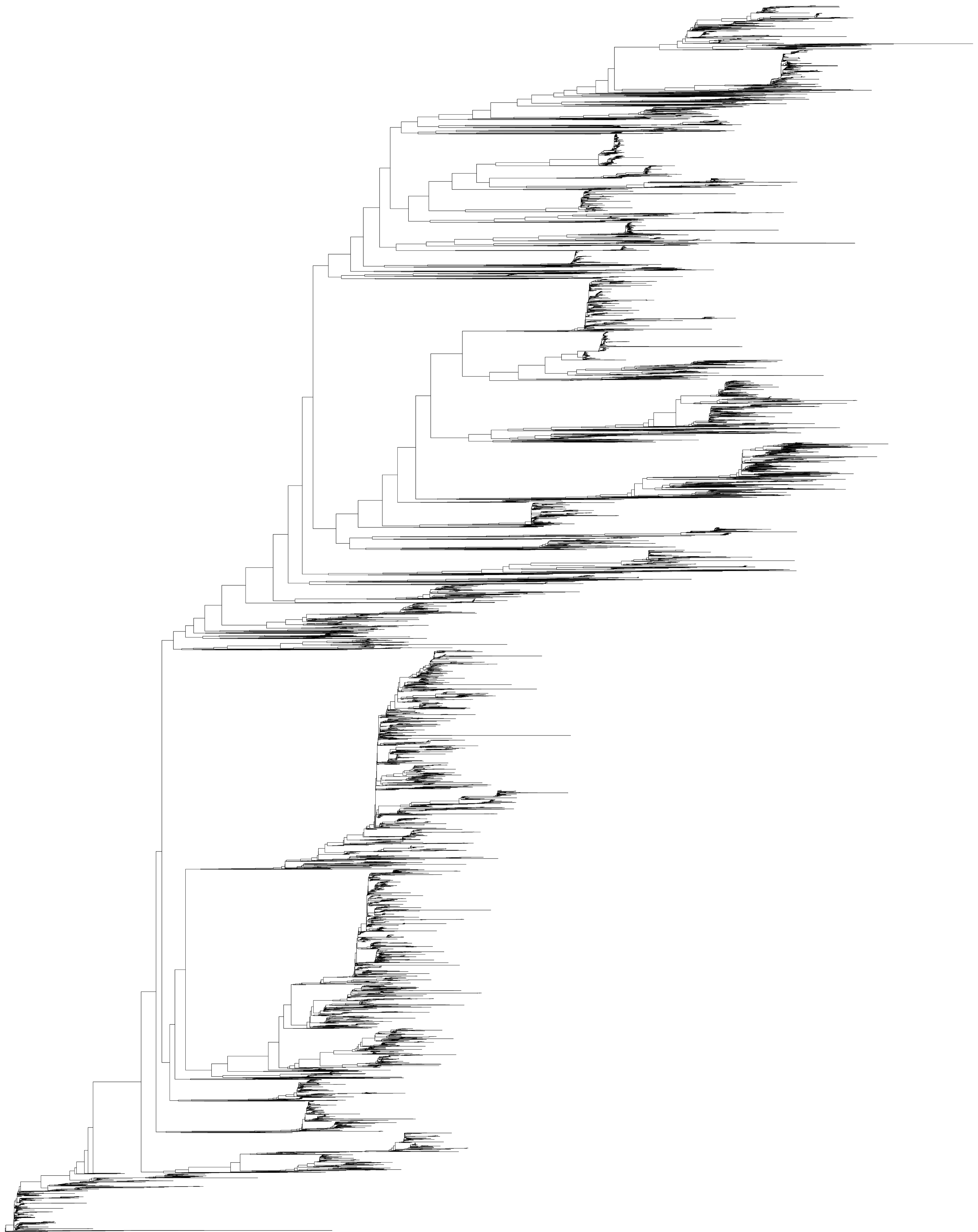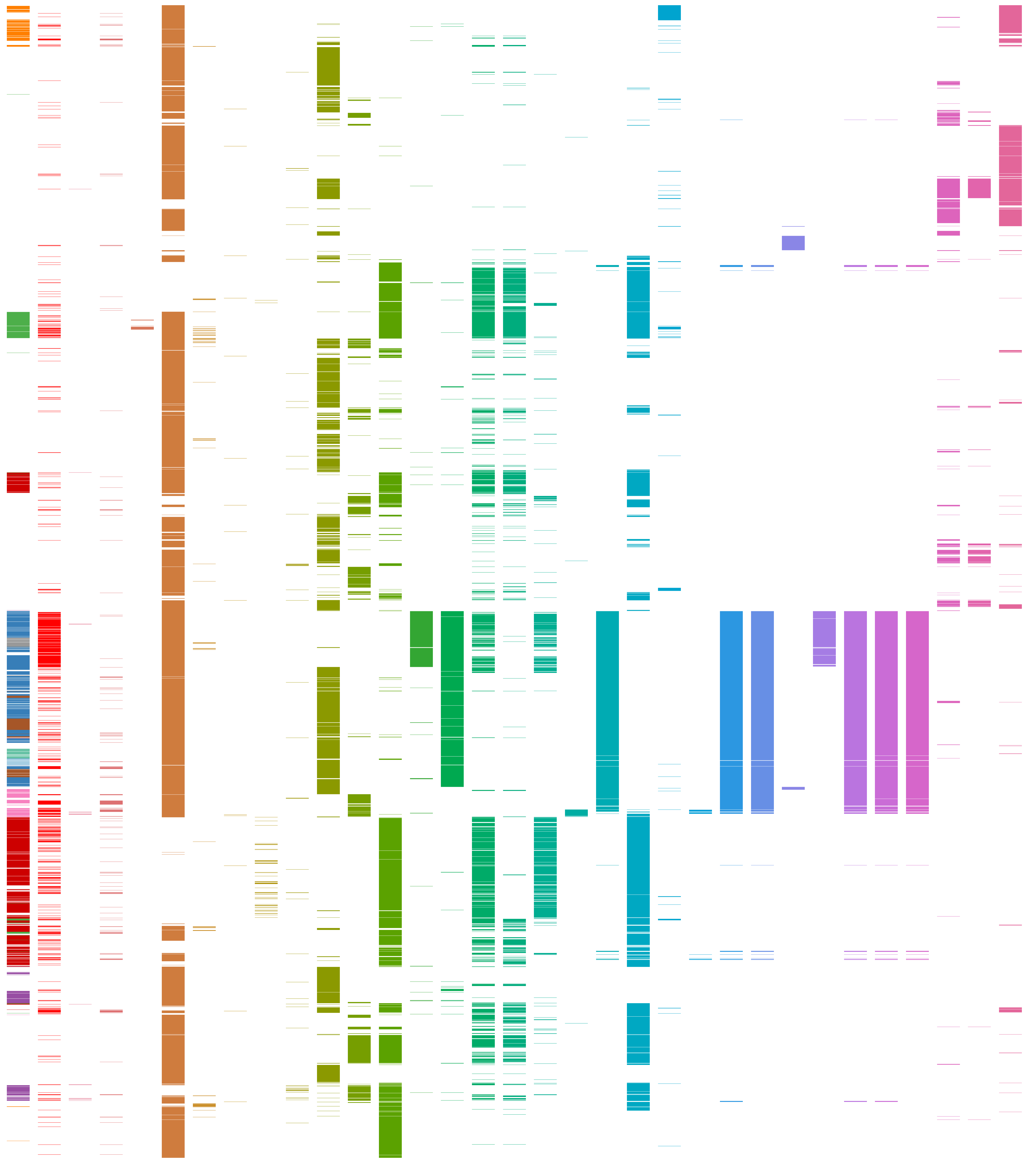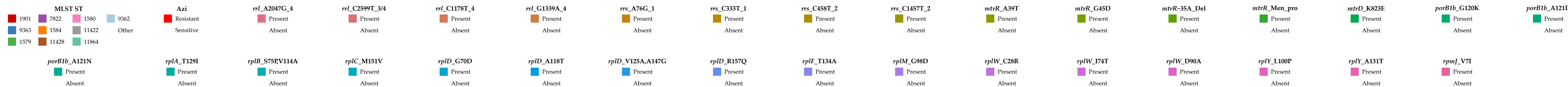

Supplement: Supplementary file 1 [file ijms-27-02258-s001.zip › Supplementary Figure S1.pdf]
